# On COVID-19 Modelling

**DOI:** 10.1365/s13291-020-00219-9

**Published:** 2020-06-29

**Authors:** Robert Schaback

**Affiliations:** Institut für Numerische und Angewandte Mathematik, Universität Göttingen, Lotzestraße 16-18, 37083 Göttingen, Germany

**Keywords:** Epidemiology, SIR model, Ordinary differential equations, 92D30, 92D25, 93C15, 34A34

## Abstract

This is an analysis of the COVID-19 pandemic by comparably simple mathematical and numerical methods. The final goal is to predict the peak of the epidemic outbreak per country with a reliable technique. The difference to other modelling approaches is to stay extremely close to the available data, using as few hypotheses and parameters as possible.

For the convenience of readers, the basic notions of modelling epidemics are collected first, focusing on the standard SIR model. Proofs of various properties of the model are included. But such models are not directly compatible with available data. Therefore a special variation of a SIR model is presented that directly works with the data provided by the Johns Hopkins University. It allows to monitor the registered part of the pandemic, but is unable to deal with the hidden part. To reconstruct data for the unregistered Infected, a second model uses current experimental values of the infection fatality rate and a data-driven estimation of a specific form of the recovery rate. All other ingredients are data-driven as well. This model allows predictions of infection peaks.

Various examples of predictions are provided for illustration. They show what countries have to face that are still expecting their infection peak. Running the model on earlier data shows how closely the predictions follow the transition from an uncontrolled outbreak to the mitigation situation by non-pharmaceutical interventions like contact restrictions.

**Supplementary Information:**

The online version of this article (10.1365/s13291-020-00219-9) contains supplementary material, which is available to authorized users.

## Introduction and Overview

During an epidemic outbreak like COVID-19, everybody wants to know how hard the impact will be. In particular:

- What is the health risk for me, my family, our friends, the city, the country, and the world?
- Is the health system prepared properly?
- Should households fill up their reserves in time?

This is a situation that asks for mathematics, like in the old times when mathematicians were needed to predict floods or solstices. Such predictions should be based on data and arguments, and they should provide well-supported suggestions for what to do. To understand the process and to make predictions, it should be modelled, and the model should be computable. Then predictions will be possible, and reality will decide later whether the model and the predictions were useful. Many models are possible, and the approach presented here is just one of them. The specific goal is to stay as close as possible to the available data, but it turns out that the available data are not directly usable for the standard models that give the basic understanding. To this end, two extensions to the standard SIR model are developed that get closer to the available data and finally are able to make data-driven predictions.

The beginning is made in Sect. 2 with an introduction to standard terms like *Basic Reproduction Number*, *Herd Immunity Threshold*, and *Doubling Time*, together with some critical remarks on their use in the media. These notions are based on the standard SIR model for epidemics that is treated in quite some detail, including proofs for most of the mathematical properties. Experts can skip over this completely. Readers interested in the predictions should jump right away to Sect. 5. For simplicity, the presentation ignores all delay-related issues like *incubation period* and *serial interval*.

To bridge the gap between model and data, Sect. 3 describes the Johns Hopkins data source with its limitations and flaws, and then presents a variation of a SIR model that can be applied directly to the data. It allows to estimate basic parameters, including the Basic Reproduction Number. But since the Johns Hopkins data provide no information about the unregistered cases and the Susceptibles, the model cannot yield reliable predictions of peaks of epidemics.

Therefore Sect. 4 combines the data-compatible model of Sect. 3 with a SIR model dealing with the unknown Susceptibles and the unregistered Infectious. This needs extra parameters that must be extracted from the literature. The first is the *infection fatality rate*, as provided e.g. by an der Heiden/Buchholz [9], Streeck et al. [20], Verity et al. [21]. Section 4.3.1 pairs it with the *case fatality rate* and shows how the latter can be deduced from the Johns Hopkins data. Like in Bommer/Vollmer [1], their combination gives a detection rate for the confirmed cases.

Sect. 4.4 introduces the second additional parameter: a recovery rate that can be directly used in the model and estimated from the infection fatality rate and the observable case fatality and case death rates. However, this parameter is not needed for prediction, just for determination of the unknown variables from the known data as long as the latter are available.

Then Sect. 5 combines all of this into a larger model that makes predictions under the assumption that there are no further changes to the parameters by political action. It estimates the parameters of a full SIR model from the available Johns-Hopkins data by the techniques of Sect. 4, using two additional technical parameters: the number of days used backwards for estimation of constants, and the number of days in which recovery or death can be expected on average, for estimation of case fatality and recovery rates. This is where time delays enter, but not into the model, only into internal estimation procedures. After the data-driven estimation of these parameters, the prediction uses only the infection fatality rate. All other ingredients are derived from the Johns Hopkins data.

Results are presented in Sect. 5. Given the large uncertainties in the Johns-Hopkins data, the predictions are rather plausible. However, reality will have the final word on this prediction model.

The paper closes with a summary and a list of open problems.

## Classical SIR Modelling

This contains the basic notions for modelling epidemics, defined and explained in mathematical terms. In particular, there will be a rigid mathematical underpinning of what is precisely meant when media talk about

- *flattening the epidemic outbreak (mitigation)*,
- *basic reproduction number*,
- *Herd Immunity Threshold*, and
- *doubling time*,

pointing out certain abuses of these notions. This will not work without calculus, but things were kept as simple as possible. Readers from outside the mathematics community should take the opportunity to brush up their calculus knowledge. Experts should go over to Sect. 3.

### The Model

The simplest standard *SIR* model of epidemics, due to Kermack-McKendrick [12] in 1927 and easily retrievable from the Wikipedia [23], deals with three variables

- Susceptible ($S$), Infectious ($I$), and Removed ($R$).

The Removed cannot infect anybody anymore, being either dead or immune. This is the viewpoint of bacteria or viruses. The difference between death and immunity of subjects is totally irrelevant for them: they cannot proliferate anymore in both cases. The SIR model cannot say anything about death rates of persons.

The Susceptible are not yet infected and not immune, while the Infectious can infect Susceptibles. Individuals move by infection from $S$ to $I$, and by death or healing from $I$ to $R$. The three classes $S$, $I$, and $R$ are disjoint and add up to a fixed total population count $N=S+I+R$. All of these are ideally assumed to be smooth functions of time $t$, and satisfy the differential equations

1 $$ \textstyle\begin{array}{rcl} \dot{S}&=&-\beta \displaystyle {\frac{S}{N} }I, \\ \dot{I}&=&+\beta \displaystyle {\frac{S}{N} }I-\gamma I, \\ \dot{R}&=&\gamma I, \end{array} $$

where the dot stands for the time derivative, and where $\beta $ and $\gamma $ are positive parameters. The product $\frac{S}{N}I$ models the probability that an Infectious meets a Susceptible and is actually infected.

Managing an SIR epidemic means *modifying* the constants $\beta $ and $\gamma $. This is why one should see the parameters as control variables, and we shall treat them even as time series from Sect. 3 on.

Note further that the Removed of the SIR model are not the Recovered of the Johns Hopkins data that we treat later, and the SIR model does not account for the Confirmed counted there. Similarly, there is no direct relation to the data published by the Robert Koch Institute. It is a major problem to match models with the available data, and we shall explain the latter to some detail in Sect. 3. The inventors Kendrick and McKermack fitted their model already in 1927 [12] to data from the plague in Bombay 1905-1906.

### Other Models

In many publications concerning COVID-19 (e.g. an der Heiden/Buchholz [9], Dandekar/Barbasthatis [2], De Brouwer et al. [3], Friston et al. [6], Khailaie et al. [13], Kucharski et al. [14], Maier/Brockmann [15]), the SIR model is extended by Exposed $E$ that are infected, but not (yet) infectious. This introduces an additional parameter and would require dealing with a latency delay properly. We avoid this complication to keep the model as simple as possible. Note that there are extensions of SIR models with 14 to 21 parameters, e.g. Friston et al. [6], Giordano et al. [7], Khailaie et al. [13]. Fitting model parameters in the above papers is partially done numerically and partially by Bayesian approaches using Markov chain sampling of prior distributions. Here, we avoid fitting and time delays as far as possible.

Conceptually different are the agent-based model that is used by Ferguson et al. [5] for parameter estimation, and the approach of Mohring et al. [16] working consistently with time delays.

### Simple Properties of the SIR Model

Since $\dot{N}= \dot{S}+ \dot{I}+\dot{R}=0$ holds in (1), the equation $N=S+I+R$ is kept valid at all times. The term $\beta \frac{S}{N}I$ moves Susceptibles to Infectious, while $\gamma I$ moves Infectious to Removed. Thus $\beta $ represents an *infection rate* while the *removal rate* $\gamma $ accounts for either healing or fatality after infection, i.e. immunity. Political decisions about reducing contact probabilities will affect $\beta $, while $\gamma $ resembles the balance between the medical aggressivity of the infection and the quality of the health care system.

As long as the Infectious $I$ are positive, the Susceptibles $S$ are decreasing, while the Removed $R$ are increasing. Excluding the trivial case of zero Infectious from now on, the Removed and the Susceptible will be strictly monotonic. Therefore we can use them to re-parameterise the model at certain places.

The SIR model is not really dependent on the total population $N$. Moreover, if we scale time by $\tau :=t\cdot \gamma $ and go over to *relative* quantities

$$ \textstyle\begin{array}{rcl} {s}(\tau )&:=&\displaystyle {\frac{S(\tau /\gamma )}{N} }, \\ {r}(\tau )&:=& \displaystyle {\frac{R(\tau /\gamma )}{N} }, \\ {i}(\tau )&:=& \displaystyle {\frac{I(\tau /\gamma )}{N} }, \end{array} $$

we get the new system

2 $$ \textstyle\begin{array}{rclcl} s'(\tau )=\displaystyle {\frac{d{s}}{d\tau } } &=& -\displaystyle { \frac{\beta }{\gamma } }{s}(\tau ){i}(\tau ) &=&-R_{0}{s}(\tau ){i}( \tau ) \\ i'(\tau )=\displaystyle {\frac{d{i}}{d\tau } } &=& \left ( \displaystyle {\frac{\beta }{\gamma } }{s}(\tau )-1\right ){i}(\tau ) &=& \left (R_{0}{s}(\tau )-1\right ){i}(\tau ) \\ r'(\tau )=\displaystyle {\frac{d{r}}{d\tau } } &=& {i}(\tau ) \end{array} $$

only containing the *Basic Reproduction Number*

3 $$ R_{0}:=\displaystyle {\frac{\beta }{\gamma } } $$

that will turn out to be of central importance. Both $\beta $ and $\gamma $ vary under a change of time scale in (1), but the basic reproduction number is invariant. Physically, $\beta $ and $\gamma $ have the dimension $\mathit{time}^{-1}$, but $R_{0}=\beta /\gamma $ and the new “time” parameter $\tau $ in (2) are dimensionless. Another interpretation of (2) is that after a time scale one can assume $\gamma =1$ and $R_{0}=\beta $. We call $\tau $ the *unit removal parameter*, because its unit can be seen as the average time needed to get removed, i.e. either dead or immune. We use a prime to denote derivatives with respect to $\tau $. But in all later sections that make real-world interpretations, we have to use real time, and then we shall go back to (1).

A standard mathematical trick is to divide the first equation by the third to get

4 $$ \textstyle\begin{array}{rcl} \displaystyle {\frac{d{s}}{d{r}} }&=&-R_{0}{s}, \\ {s}({r})&=&{s}({r}(0))\exp (-R_{0}({r}-{r}(0))). \end{array} $$

We shall use (4) in Sect. 2.11 to study the long-term behaviour of solutions. The introduction of (4) is a typical pitfall for mathematics: it is a nice theoretical simplification, but it obscures the most interesting practical aspect, in this case the fraction $i$ of infectious persons in the population. The same holds for the simplification by setting $d\tau =\gamma \frac{I}{N}dt$ that is ignored here, leaving it to interested readers.

### Examples

**Fig. 1:**
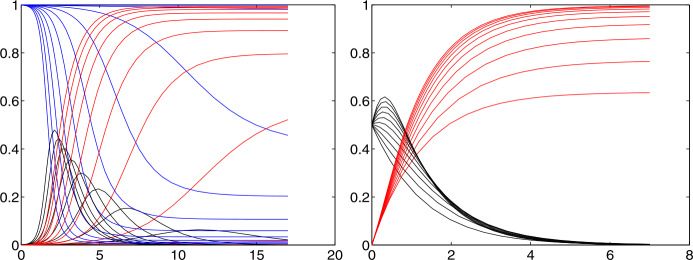
Some typical SIR system solutions, relative to the total population. See the explanation in Sect. 2.4. The peaked curves for the Infectious are “flattened” for small $R_{0}$

Figure 1 shows a series of test runs of a SIR model. Recall that the relative Recovered $r$ are increasing from zero, and the relative Susceptibles $s$ are decreasing down from one. The relative Infectious $i$ are in between and can possibly show a sharp peak that everybody tries to avoid. We shall deal with the mathematics of the peak in Sects. 2.8, 2.13, and 2.14, while the rest of the paper focuses on data-driven predictions of peaks. The Infectious are usually not covered by the media who tend to focus on the cumulative number of confirmed cases, containing the Removed.

In both plots we set $r(0)=0$, $\gamma =1$, and let $R_{0}=\beta $ vary from 0.1 to 5. The difference between the figures lies in the initial value $i(0)$. Left, due to a realistically small $i(0)=0.001$, one cannot see the decaying peak-less cases of $i$ near startup for $R_{0}<1$, while the right-hand plot has $i(0)=1/2$ and shows them. Decreasing $R_{0} \searrow 1$ flattens the peaks of the Infectious $i$, and there is no peak for $R_{0}\leq 1$. Furthermore, one can observe that $i$ always decays to zero, while $s$ and $r$ tend to fixed positive levels in the long run. The final level of $r$ is particularly interesting because part of it is the total death toll. It decreases when $R_{0}$ decreases. We shall prove all of this later. When countries change parameters by administrative actions like a shutdown, they jump to a more flat $i$ curve, e.g. at an intersection point.

From the system, one can also infer that $r$ has an inflection point where $i$ has its maximum, since $r''=i'$. If only $r$ would be observable, one could locate the peak of $i$ via the inflection point of $r$. Finally, note that small initial values $i(0)$ of $i$ delay the peak considerably, no matter how large $R_{0}$ is. We shall prove this in Sect. 2.14.

### Interpretation of the Basic Reproduction Number $R_{0}$

Media often say that $R_{0}$ gives the number of persons an average Infectious infects while being infectious. This is a rather mystical statement that needs underpinning. In the SIR system (1) the quantity

$$ \displaystyle {\frac{1}{\gamma } }=\displaystyle { \frac{I}{\dot{R}} } $$

is a value that has the physical dimension of time. It describes the ratio between current Infectious and current newly Removed, and thus can be seen as the average time needed for an Infectious to get Removed, i.e. the average time that an Infectious can infect others. This is why we called the dimensionless $\tau =t\cdot \gamma $ the *unit removal parameter* in Sect. 2.3. Correspondingly,

$$ \dot{I}+\gamma I= \dot{I} +\dot{R} =\beta \displaystyle {\frac{S}{N} }I $$

are the newly Infected, and therefore

$$ \displaystyle {\frac{1}{\beta } }\displaystyle {\frac{N}{S} }= \displaystyle {\frac{I}{\dot{I} +\dot{R}} } $$

can be seen as the time it needs for an average Infectious to generate a new Infectious. The ratio $R_{t}:=\frac{\beta }{\gamma }\frac{S(t)}{N}$ then gives how many new Infectious can be generated by an Infectious while being infectious. This is the time-dependent *Reproduction Number*, but it is only close to $R_{0}$ if $S(t)\approx N$, i.e. at the start of an outbreak. A correct statement is that $R_{0}$ is the average number of infections an Infectious generates while being infectious, but within an unlimited supply of Susceptibles.

To let less new Infectious be generated, administrative actions try to change the parameters of the epidemic towards small $R_{0}$. We shall see that this is correct from a mathematical viewpoint as well, and we shall study the influence of $R_{0}$ to quite some detail.

The above interpretation of $R_{0}$ shows two major ways to make $R_{0}$ small: reducing the number of possibly infective contacts, and reducing the time an Infectious has to infect others. The second works by putting all infectious persons into strict quarantine, while first can be done by reducing contacts of all persons, even the Susceptibles, and reducing the infection probability for each contact, e.g. by wearing masks.

SIR-based models of the COVID-19 pandemics estimate $R_{0}$ between 2 and 6 during an uncontrolled outbreak (see e.g. the Robert Koch-Institute [17], De Brouwer et al. [3], Dehning et al. [4], and Maier/Brockmann [15]), while *non-pharmaceutical interventions* (NPI) bring $R_{0}$ below 1. We shall see examples in 3.3.2 and 5.2.

The use of the Basic Reproduction Number $R_{0}$ in the media suggests that large $R_{0}$ are generally serious, because each Infectious infects several people. This is only true at the beginning of an outbreak, because then there are enough Susceptibles. But it will turn out in Sect. 2.8 that the Infectious will always finally go to zero, whatever the Basic Reproduction Number is. See Fig. 1 as well.

### Conditions for Outbreaks

The first interesting question in a beginning epidemic is:

- Will there be a serious outbreak, or will the infection disappear quickly?

Therefore we first look at the initial conditions for the model. Since everything is invariant under an additive time *shift*, we can start at time 0, and since time *scales* are irrelevant to the problem at startup, we can use the simplified system (2).

The relative Infectious $i$ in (2) do not increase right from the start if $\dot{I}(0)\leq 0$, i.e.

5 $$ s(0)\leq \displaystyle {\frac{1}{R_{0}} }, $$

and then they decrease further since the Susceptibles $s$ must decrease and

6 $$ \displaystyle {\frac{i(\tau )'}{i(\tau )} }=(\log i(\tau ))'= R_{0} s( \tau )-1< R_{0}s(0)-1\leq 0. $$

There is no outbreak, and this must occur for all initial conditions if $R_{0}\leq 1$. But if $R_{0}>1$, the outbreak depends on the initial condition (5). Altogether, outbreaks are fully characterised by

7 $$ 1>s(0)>\displaystyle {\frac{1}{R_{0}} }. $$

### Herd Immunity Threshold

In connection with an outbreak, the *Herd Immunity Threshold*

$$ \mathit{HIT}=1-\displaystyle {\frac{1}{R_{0}} } $$

is often mentioned. The background question is:

- If an uninfected population is threatened by an infection with Basic Reproduction Number $R_{0}$, what is the number of immune persons needed to prevent an outbreak right from the start?

In the idealised situation $i(0)=0$ and $s(0)+r(0)=1$,

$$ r(0)=1-\displaystyle {\frac{1}{R_{0}} }=\mathit{HIT} $$

follows from (5) and (7) as the threshold between outbreak and decay for the relative Removed. This does not refer to a whole epidemic scenario. It is to be checked *before* anything happens, and useless within a developing epidemic, whatever the media say.

### The Peak

In the outbreak case (7), the main questions are:

- When will the Infectious reach their maximum?
- How large will the maximal value be?

More generally, we ask for a time $t_{I}$ or a unit removal parameter $\tau _{I}=\gamma \,t_{I}$ where the Infectious $i$ are positive and do not change. Then we have

8 $$ 0=\displaystyle {\frac{di}{d\tau } }(\tau _{I})=(R_{0}s(\tau _{I})-1)i( \tau _{I}), $$

and the monotonicity of $s$ implies uniqueness of $\tau _{I}$ and

9 $$ s(\tau _{I})=\displaystyle {\frac{1}{R_{0}} }. $$

If $i$ would increase without reaching a maximum in finite time, the first equation of (2) would imply that $s$ goes exponentially to zero, but then there is a $\tau _{I}$ with (9), and (8) follows. Summarising, this proves that whenever there is an outbreak by (7), there is a unique maximum of the relative Infectious $i$ that we call the *peak* from now on. Behind the peak, or apart from any outbreak situation, the Infectious must go exponentially to zero due to (6), because the Susceptibles continue to decrease, no matter how large $R_{0}$ is.

Determining the peak is theoretically difficult, and in practice it requires good estimates for $\beta $ and $\gamma $. Mathematical results on the peak will be in Sects. 2.13 and 2.14, while data-driven predictions follow in Sect. 5.2

In real life it is highly important to avoid the peak situation, and this can only be done by administrative measures that change $\beta $ and $\gamma $ in (1) to the situation $\beta <\gamma $. This is what management of epidemics is all about, provided that an epidemic follows the SIR model. We shall see how countries perform.

In the peak situation of (8) and (9), the fraction

10 $$ 1-\displaystyle {\frac{1}{R_{0}} } = 1-s(\tau _{I})=r(\tau _{I})+i( \tau _{I})\geq i(\tau _{I}) $$

of the relative Non-Susceptible at the peak is exactly the Herd Immunity Threshold. Thus it is correct to say that if the Immune of a population are below the Herd Immunity Threshold at startup, and if the Basic Reproduction Number is larger than one, the sum of the Immune and the Infectious will rise up to the Herd Immunity Threshold and then the Infectious will decay. This is often stated imprecisely in the media.

### Analysing the Outbreak

When an outbreak starts, almost everybody is susceptible, i.e. $s(0)\approx 1$, and then

$$ i'=R_{0}s-1\approx R_{0}-1 $$

models an exponential outbreak with exponent $R_{0}-1>0$ in unit removal parametrisation, with a solution

$$ i(\tau )\approx i(0)\exp ((R_{0}-1)\tau ). $$

If this is done in real time $t$ and discrete time steps $\Delta t$, the system (1) yields

$$ \displaystyle {\frac{I(t+\Delta t)}{I(t)} }\approx \exp ((\beta - \gamma )\Delta t). $$

The severity of the outbreak in real time is not controlled by $R_{0}=\beta /\gamma $, but rather by $\beta -\gamma $. Publishing single values $I(t)$ does not give any information about $\beta -\gamma $. Better is the ratio of two subsequent values

11 $$ \displaystyle {\frac{I(t_{2})}{I(t_{1})} }\approx \exp ((\beta - \gamma )(t_{2}-t_{1})), $$

and if this gets smaller over time, the outbreak gets less dramatic because $\beta -\gamma $ gets smaller. But (11) is by mo means a correct way to estimate $R_{0}$.

Therefore, really useful information about an outbreak must concern $I$, but should not consist of single values. Increments in percent are much better, because their logarithm is proportional to $\beta -\gamma $. However, it needs increments of increments to see whether administrative actions are successful by changing $\beta -\gamma $. This is what the media rarely provided during the outbreak. On the positive side, the severity of a future outbreak in unit removal parametrisation is described correctly by estimates of $R_{0}>1$, if these have a solid mathematical and experimental basis. All changes of $R_{0}$ should be carefully monitored.

### Doubling Time

Another information used by media during an outbreak is the *doubling time*, i.e. how many days it takes until daily values double. It is $n\Delta t$ with the number $n$ from

$$ 2=\displaystyle {\frac{I(t+n \Delta t)}{I(t)} } \approx \exp (( \beta -\gamma )n\Delta t)=(\exp ((\beta -\gamma )\Delta t)^{n} $$

or

$$ n=\displaystyle {\frac{\log 2}{(\beta -\gamma )\Delta \tau } }, $$

i.e. it is inversely proportional to $\beta -\gamma $. If political action doubles the doubling time, if halves $\beta -\gamma $. If politicians do this repeatedly, they never reach $\beta <\gamma $, and they never escape an exponential outbreak if they do this any finite number of times. Extending the doubling time will never prevent a peak, it only postpones it and hopefully flattens it. When presenting a doubling time, media should always point out that this makes only sense during an exponential outbreak. And it is not related to the basic reproduction number $R_{0}=\beta /\gamma $, but to the difference $\beta -\gamma $.

### Long-Term Behaviour

Aside from the peak, it is interesting to know the portions of the population that get either permanently removed (by death or immunity) or never come into contact with the infection. This concerns the long-term behaviour of the Removed and the Susceptibles. Figure 1 demonstrates how $r$ and $s$ level out under all circumstances shown, but is this always true, and what is the final ratio? And if one has additional information on the percentage of casualties within the Removed, what is the total death toll in the long run?

Going back to (4), we get

12 $$ {s}({r})={s}(0)\exp \left (-R_{0}{r}\right ) $$

when assuming $r(0)=0$ at startup. Since ${r}$ is increasing, it has a limit $0<{r}_{\infty }\leq 1$ for $\tau \to \infty $, and in this limit

$$ s_{\infty }={s}(0)\exp \left (-R_{0}{r}_{\infty }\right ) $$

holds, together with the condition ${r}_{\infty }+s_{\infty }=1$, because there are no more Infectious. The transcendental equation

13 $$ {s}(0)\exp \left (-R_{0}{r}_{\infty }\right )=1-{r}_{\infty }$$

has a unique solution in $(0,1)$ dependent on ${s}(0)<1$ and $R_{0}$. Therefore the Infectious always go to zero, but Susceptibles always remain. Then a new infection can always arise as soon as an infected person enters the sanitised population. The outbreak risk is dependent on the portion ${s}_{\infty }=1-{r}_{\infty }$ of the Susceptibles by (5). This illustrates the importance of vaccination, e.g. against measles or influenza.

**Fig. 2:**
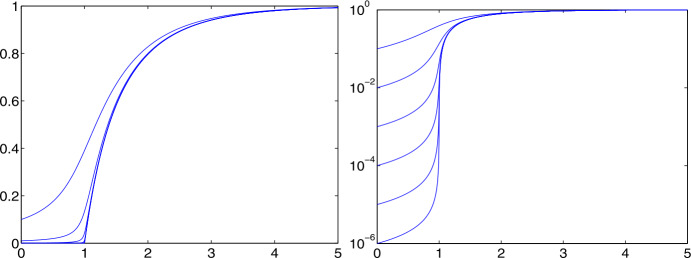
The asymptotic level $r_{\infty }$ of the relative Removed as a function of $R_{0}$ for $s(0)=0.9,\,0.99, \,0.999$ etc. as curves from the top. Right: logarithmic scale

To see how $r_{\infty }$ and $s_{\infty }=1-r_{\infty }$ behave as functions of $R_{0}$ and $s(0)$, we solve the equation (13) by the Lambert $W$ function to get

14 $$ r_{\infty }=1+\displaystyle {\frac{1}{R_{0}} }W\left (-s(0)R_{0}\exp (-R_{0}) \right ) $$

with a surprising behaviour. See Fig. 2 for illustration. Left, the curves for unrealistically small initial values $s(0)=0.9,\;0.99$ and 0.999 for Susceptibles can still be distinguished from the more interesting curves below that coincide for all $s(0)$ closer to one and have a sharp turn at $R_{0}=1$. The logarithmic plot to the right shows that for $R_{0}<1$ the curves separate, and that it pays off significantly to have $R_{0}<1$ for $s(0)$ close to one.

This has some serious implications, if the model is correct for an epidemic situation. When politicians try to “flatten the curve” by bringing $R_{0}$ below 1 at some early time when the Susceptibles are still abundant, the asymptotic rate $r_{\infty }$ of Removed will be *dramatically* smaller than for any other situation, because one stays left of the sharp turn in Fig. 2. This is particularly important if the rate of fatalities within the Removed is high.

Large values of $R_{0}$ lead to large relative values of Removed to Susceptible in the limit. The consequence is that systems with large $R_{0}$ have a dramatic outbreak and lead to a large portion of Removed. This is good news if the rate of fatalities within the Removed is low, but very bad news otherwise. When pressing $R_{0}$ below one, the risk of re-infection rises due to the larger portion of Susceptibles, but the deaths contained in the Removed are kept low.

The decay situation (5) implies that ${s}_{\infty }\leq {1}/{R_{0}} $ holds, and consequently

$$ {r}_{\infty }=1-{s}_{\infty }\geq 1-\displaystyle {\frac{1}{R_{0}} }=\mathit{HIT}. $$

Therefore the final rate of the Removed is not smaller than the Herd Immunity Threshold. This is good news for possible re-infections, but only if the death rate among the Removed is small enough.

### Asymptotic Exponential Decay

If we go back to (6) for a unit removal parameter $\tau _{D}$ where $i$ decreases, in an outbreak or not, we have $R_{0} s_{\infty }\leq R_{0}{s}(\tau _{D})<1 $ and then

$$ i(\tau _{D})\exp ((R_{0}{s}_{\infty }-1)(\tau -\tau _{D}))\leq i(\tau ) \leq i(\tau _{D})\exp ((R_{0}s(t_{D})-1)(\tau -\tau _{D})) $$

for all $\tau \geq \tau _{D}$. Therefore the exponential decay in unit removal parametrisation is not ruled by $R_{0}-1$ as in the outbreak case with $R_{0}>1$, but rather by $R_{0}{s}_{\infty }-1$. This also holds for large $R_{0}$ because ${s}_{\infty }$ counteracts. The bell shapes of the peaked $i$ curves are not symmetric with respect to the peak. Inserting (14), the relative Infectious always decay asymptotically exponentially like

$$ \exp ((R_{0}s_{\infty }-1)\tau )=\exp ((W(-s(0)R_{0}\exp (-R_{0}))-1) \tau ) \text{ for } \tau \to \infty $$

with the Lambert $W$ function. By MAPLE, the slowest decay arises for $R_{0}=1$.

### Maximal Infectious at the Peak

At the peak of the Infectious $i$ at $\tau _{I}$ in an outbreak (7) with $r(0)=0$ we know

$$ {s}(\tau _{I})=\displaystyle {\frac{1}{R_{0}} }={s}({r}(\tau _{I})) ={s}(0) \exp \left (-R_{0}{r}(\tau _{I})\right ) $$

from (9) and (4), and get the Removed at the peak as

15 $$ {r}(\tau _{I})=\displaystyle {\frac{1}{R_{0}} }\log ({s}(0)R_{0}). $$

Then the exact value of the Infectious $i$ at the peak is

16 $$ i(\tau _{I})=1-{s}(\tau _{I})-{r}(\tau _{I})= 1-\displaystyle { \frac{1}{R_{0}} }-\displaystyle {\frac{1}{R_{0}} }\log ({s}(0)R_{0}), $$

improving (10). Note that the log is positive due to the outbreak condition (7). It is remarkable that the *value* of $i$ at the peak does not depend on initial conditions, while the next section proves that the *position* of the peak does.

For standard infections that have starting values ${s}(0)=S(0)/N$ very close to one, the maximal ratio of Infectious is

$$ i(\tau _{i}) \approx 1-\displaystyle {\frac{1}{R_{0}} }- \displaystyle {\frac{1}{R_{0}} }\log (R_{0}). $$

**Fig. 3:**
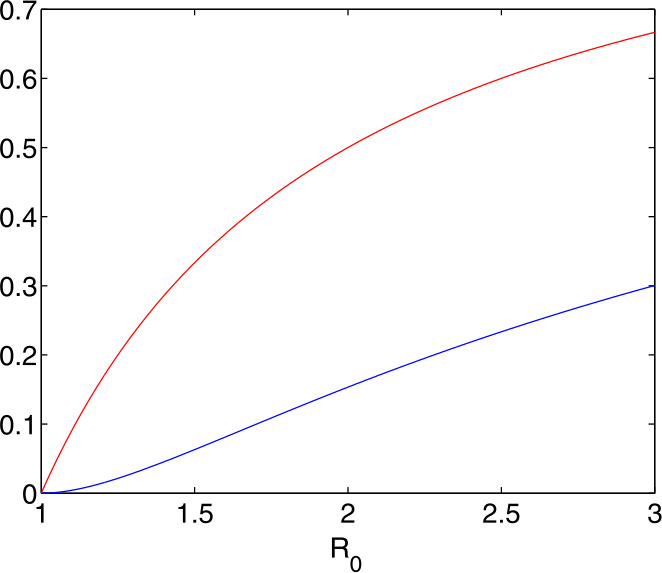
The effect of $R_{0}$ on the peak value $i(\tau _{i})$ of Infectious

Figure 3 shows the behaviour of this function, as the lower curve. A value of $R_{0}=4$ leads to a maximum of more than 40% of the population infectious at a single time. If 5% need hospital care, a country needs hospital beds for 2% of the population around peak time. This disaster calls for mitigation by lowering $R_{0}$.

The upper curve leaves the log term out, i.e. it marks the rate (9) of the Susceptibles at the peak, and by (10) the difference is the rate $r(\tau _{i})$ of the Recovered at the peak. It also marks the extreme case in (7) with $R_{0}s(0)=1$, i.e. having the smallest possible initial value of $s(0)$ for a given $R_{0}$ to generate an outbreak. Therefore all $s(0)$-dependent possibilities vary between the two curves.

### Localising the Peak

Knowing now how large the peak is, we want to find out where it is. We write the unit removal parameter $\tau $ as a function of $r$ by $\frac{d\tau }{dr}=(\frac{dr}{d\tau })^{-1}=\frac{1}{i}$ and integrate from $r=0=r(0)$ to $r=r(\tau _{I})$ to get the peak position

$$ \tau _{I}=\int _{0}^{r(\tau _{I})}\frac{1}{i(r)}dr =\int _{0}^{\log (s(0)R_{0})/R_{0}} \frac{1}{1-r-s(0)\exp (-R_{0}r)}dr $$

as a nasty function of $s(0)$ and $R_{0}$, using (2), (12), and $1=i(r)+s(r)+r$. To prove that the peak moves towards zero for both limits $R_{0}\nearrow \infty $ and $R_{0}\searrow 1$, we first observe that $i\geq i(0)$ holds left of the peak. Then we use (15) to get

17 $$ \tau _{I}\leq \displaystyle {\frac{r(\tau _{i})}{i(0)} } = \displaystyle {\frac{1}{i(0)R_{0}} }\log (s(0)R_{0})\leq \displaystyle {\frac{1}{i(0)R_{0}} }\log (R_{0})\leq \displaystyle { \frac{1}{e\cdot i(0)} }\approx \displaystyle {\frac{0.37}{i(0)} } $$

by inserting the maximum of $\log (R_{0})/R_{0}$ at $e$. The upper bound gets large when $i(0)$ gets small, a realistic case by Figs. 1 and 8. This calls for a lower bound.

For fixed $s(0)$ and $i(0)$ there will be a maximal peak position for a rather specific $R_{0}$. A MAPLE-based analysis shows that $R_{0}(s(0))=-W(-s(0)/e)^{-1}$ with Lambert’s $W$ function yields

$$ \tau _{I}\geq \displaystyle {\frac{0.3\,(1-i(0))}{\sqrt{i(0)}} }. $$

Therefore the peak can indeed move arbitrarily far out for small $i(0)$ and large $s(0)=1-i(0)$. There is not much leeway for smaller $R_{0}$ to bring the peak position to zero for large $s(0)$, namely $\frac{1}{s(0)}< R_{0}< R_{0}(s(0))$. Both bounds for $R_{0}$ tend to one for $s(0)\to 1$.

The practical consequence is that keeping $R_{0}>1$ close to one by mitigation is no good idea, because the peak can move far into the future for realistically small $i(0)$, delaying the epidemic in an intolerable way. Countries should go for $R_{0}$ considerably smaller than one.

### Turnaround Time

In a peak situation (7) one can consider the *turnaround parameter* $\tau _{T}$ at which the Infectious $i$ come back to their starting value $i(0)$ behind the peak. At that point the population has accumulated more Removed, dead or immune. We calculate the integral

$$ \int _{0}^{\infty }i(\tau )d\tau =\int _{0}^{\infty }r'(\tau )d\tau =r_{\infty }-r(0). $$

The rectangle of length $\tau _{T}$ and height $i(0)$ fits under the $i$ curve, and therefore

$$ i(0)\tau _{T}\leq r_{\infty }-r(0)\leq r_{\infty }\leq 1, $$

proving that the real turnaround *time* $t_{T}=\tau _{T}/\gamma $ has a fixed bound $t_{T}\leq r_{\infty }/(i(0)\gamma )$. From Fig. 2 one can see that making $R_{0}$ smaller will decrease the bound via $r_{\infty }$.

### Estimating and Varying Parameters

If real-time data for the SIR model (1) were fully available, one could solve for

18 $$ \textstyle\begin{array}{rclrcl} \gamma &=&\displaystyle {\frac{\dot{R}}{I} },& b&:=&\beta \displaystyle {\frac{S}{N} } = \displaystyle { \frac{\dot{I}+\gamma I}{I} }=\displaystyle {\frac{\dot{I}+\dot{R}}{I} }, \\ \beta &=&\displaystyle {\frac{N}{N-I-R} }\cdot \displaystyle { \frac{\dot{I}+\dot{R}}{I} },& R_{0}&=&\displaystyle {\frac{N}{N-I-R} } \cdot \displaystyle {\frac{\dot{I}+\dot{R}}{\dot{R}} }= -\displaystyle { \frac{N}{S} }\displaystyle {\frac{\dot{S}}{\dot{R}} } =- \displaystyle {\frac{1}{{s}} }\displaystyle {\frac{d{s}}{d{r}} }, \end{array} $$

and we shall use this in Sect. 3.3. The validity of a SIR model can be tested by checking whether the right-hand sides for $\beta $, $\gamma $ and $R_{0}$ are roughly constant. If data are sampled locally, e.g. before or after a peak, the above technique should determine the parameters for the global epidemic and be useful for either prediction or backward testing.

However, in pandemics like COVID-19, the parameters $\beta $ and $\gamma $ change over time by administrative action. This means that they should be considered as functions in the above equations, and then their changes may be used for conclusions about the influence of such actions. From this viewpoint, one can go back to the SIR model and consider $\beta $ and $\gamma $ as control functions that just describe the relation between the variables.

But the main argument against using (18) is that the data are rarely available. This is the concern of the next section.

## Using Available Data

Now we confront the modelling of the previous section with available data. This is crucial for manoeuvering countries through the epidemics (Sentker [19]).^1^ From now on we have to work in real time and go back to (1) instead of all mathematical simplifications.

### Johns Hopkins Data

We work with the COVID-19 data from the Johns Hopkins University at GitHub [8]. They are the only source that provides comparable data on a worldwide scale, namely

1. Confirmed ($C$) or *cumulative infected*
2. Dead ($D$), and
3. Recovered ($R$), i.e. alive and immune,

as cumulative integer valued time series for days from Jan. 22nd, 2020. All these values are absolute numbers, not relative to a total population. Note that the unconfirmed cases and the Susceptibles are not accessible at all, while the Confirmed contain the Dead and the Recovered of earlier days.

The media, in particular German TV, present COVID-19 data in a rather debatable way. When mentioning Johns Hopkins data, they provide $C$, $D$, and $R$ separately without stating the most important figures, namely $I=C-D-R$, their change, and the change of their change. When mentioning data of the Infectious from the Robert Koch institute alongside, they do not say precisely that these are non-cumulative and should be compared to the $I=C-R-D$ data of the Johns Hopkins University. And, in most cases during the outbreak, they did not mention the change of the change. Quite like all other media.

**Fig. 4:**
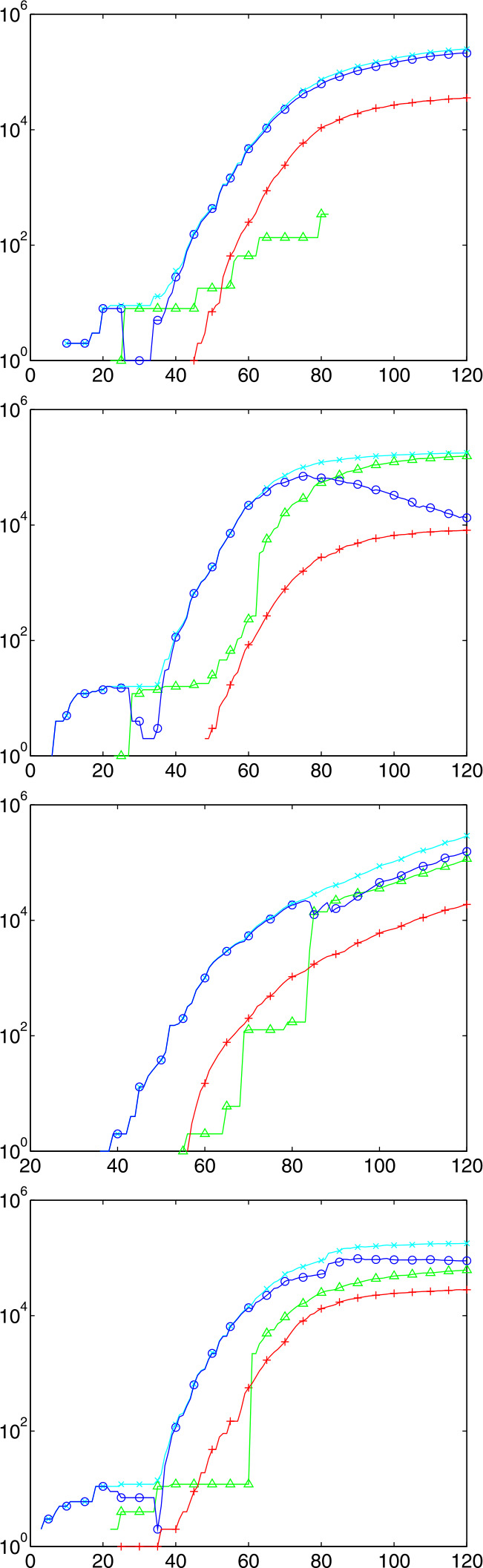
Raw Johns Hopkins data in logarithmic presentation up to day 120, from top: UK, Germany, Brazil, and France. Markers $X$ for Confirmed, $O$ for Infectious, ∧ for Recovered, + for Deaths, not on all data points

We take the data as presented, but there are many well-known flaws. In particular, the values for specific days are partly belonging to previous days, due to delays in the chains of data transmission in different countries. This is why, at some points, we shall apply some conservative smoothing of the data. Finally, there are inconsistencies that possibly need data changes. In particular, there are countries like Germany who deliver data of Recovered in a very questionable way. The law in Germany did not enforce authorities to collect data of Recovered, and the United Kingdom did not report numbers of Dead and Recovered from places outside the National Health System, e.g. from Senior’s retirement homes. Both strategies have changed somewhat in the meantime, as of early May, but the data still keep these flaws. See Fig. 4 for examples.

We might assume that the Dead plus the Recovered of the Johns Hopkins data are the Removed of the SIR model, and that the Infectious $I=C-R-D$ of the Johns Hopkins data are the Infectious of the SIR model. But this is not strictly valid, because the Johns Hopkins data concern only registered cases.

On the other hand, one can take the radical viewpoint that facts are not interesting if they do not show up in the Johns Hopkins data. Except for the United Kingdom, the important figures concern COVID-19 casualties that are actually registered as such, others do not count, and serious cases needing hospitalisation or leading to death should not go unregistered. If they do in certain countries, using such data will not be of any help, unless other data sources are available.

An important point for what follows is that the data come as daily values. To make this compatible with differential equations, we shall replace derivatives by differences.

### Examples

To get a first impression about the Johns Hopkins data, Fig. 4 shows raw data up to day 120, May 21st. For better visibility, not all data points have markers. Here, and in all plots to follow, the $x$ axis has the days after Jan. 22nd, 2020. It might be helpful to remember that day 100 is May 1st. The $y$ axis is logarithmic, because then linearly increasing or decreasing parts in the figures correspond to exponentially increasing or decreasing numbers in the real data.

Many presentations in the media are non-logarithmic, and then all exponential outbreaks look similar. The most interesting data are the Infectious $I=C-R-D$ marked by $O$ that show a peak or not, and the cumulative casualties $D$ marked by +. The data for other countries tell similar stories and are suppressed.

One can see in Fig. 4 that Germany has passed the peak of the Infectious, while France is roughly at the peak and the United States and Brazil are still in an exponential outbreak. The early figures, below day 40, are rather useless, but then an exponential outbreak is visible in all cases. This outbreak changes its slope due to political actions, and we shall analyse this later. See Dehning et al. [4] for a detailed early analysis of slope changes.

There are strange anomalies in the Recovered (∧ marker). France seems not to have delivered any data between days 40 and 58, Germany changed the data delivery policy between days 62 and 63, and the UK data for the Recovered are a mess. We shall avoid using data on the Recovered as much as possible.

It should be noted that the available medical results on the COVID-19 disease often state that Confirmed will die or survive after a more or less fixed number of days. This would imply that the curves marked + for the Dead and the curves marked ∧ for the Recovered should roughly follow the curves marked $X$ for the Confirmed with a fixed but measurable delay. This is partially observable, but much less accurately for the Recovered.

### The Johns Hopkins Data Model

We now define a model that works exclusively with the Johns Hopkins data, but comes close to a SIR model, without being able to use $S$. Since the SIR model does not distinguish between recoveries and deaths, we set in obvious notation

$$ R_{\mathit{SIR}}\Leftrightarrow D_{\mathit{JH}}+R_{\mathit{JH}} $$

and let the Infectious be comparable, i.e.

$$ I_{\mathit{SIR}} \Leftrightarrow I_{\mathit{JH}}:=C_{\mathit{JH}}-D_{\mathit{JH}}-R_{\mathit{JH}} $$

which implies

$$ (I+R)_{\mathit{SIR}}\Leftrightarrow C_{\mathit{JH}}, $$

and we completely omit the Susceptibles. From now on, we shall drop the subscript $\mathit{JH}$ when we use the Johns Hopkins data, but we shall use $\mathit{SIR}$ when we go back to the SIR model.

Now we take (18) of Sect. 2.16 and insert differences:

$$ \textstyle\begin{array}{rcl} \gamma &=&\displaystyle {\frac{\dot{R}_{\mathit{SIR}}}{I_{\mathit{SIR}}} } \\ &\approx & \displaystyle {\frac{(D+R)_{n+1}-(D+R)_{n}}{I_{n}} }=: \gamma _{n} \\ b:=\beta \displaystyle {\frac{S_{\mathit{SIR}}}{N} } &=& \displaystyle { \frac{\dot{I}_{\mathit{SIR}}+\gamma I_{\mathit{SIR}}}{I_{\mathit{SIR}}} } =\displaystyle { \frac{\dot{I}_{\mathit{SIR}}+\dot{R}_{\mathit{SIR}}}{I_{\mathit{SIR}}} }, \\ &\approx & \displaystyle {\frac{C_{n+1}-C_{n}}{I_{n}} }=:b_{n}, \end{array} $$

defining time series $\gamma _{n}$ and $b_{n}$ that model $\gamma $ and $b=\beta \cdot S_{\mathit{SIR}}/N$ without knowing $S_{\mathit{SIR}}$. This is equivalent to the model

$$ \textstyle\begin{array}{rcl} C_{n+1}-C_{n} &=& b_{n}I_{n}, \\ I_{n+1}-I_{n}&=&b_{n}I_{n}-\gamma _{n} I_{n}, \\ (R+D)_{n+1}-(R+D)_{n}-&=& \gamma _{n} I_{n} \\ \end{array} $$

that maintains $C=I+R+D$, and we may call it a *Johns Hopkins Data Model*. It is very close to a SIR model if the time series $b_{n}$ is not considered to be constant, but just an approximation of $\beta \cdot S_{\mathit{SIR}}/N$.

#### Estimating $R$

By brute force, one can take

19 $$ r_{n}=\displaystyle {\frac{b_{n}}{\gamma _{n}} }=\displaystyle { \frac{C_{n+1}-C_{n}}{R_{n+1}+D_{n+1}-R_{n}-D_{n}} } $$

as a data-driven substitute for

$$ \frac{\beta }{\gamma } \frac{S_{\mathit{SIR}}}{N}=R_{0}\frac{S_{\mathit{SIR}}}{N}. $$

Then there is a rather simple observation:

- If $r_{n}$ is smaller than one, the Infectious decrease.

It follows using (19) via

$$ \textstyle\begin{array}{rcl} I_{n+1}-I_{n}&=&C_{n+1}-C_{n}-(R_{n+1}-R_{n}+D_{n+1}-D_{n}) \\ &=&(r_{n}-1)(R_{n+1}+D_{n+1}-R_{n}-D_{n}), \end{array} $$

but this is visible in the data anyway and not of much help.

Since $r_{n}$ models $R_{0}\frac{S_{\mathit{SIR}}}{N}$, it always underestimates $R_{0}$. This underestimation gets dramatic when it must be assumed that $S_{\mathit{SIR}}$ gets seriously smaller than $N$.

At this point, it is not intended to forecast the epidemics. The focus is on extracting parameters from the Johns Hopkins data that relate to a background SIR-type model.

#### Example

**Fig. 5:**
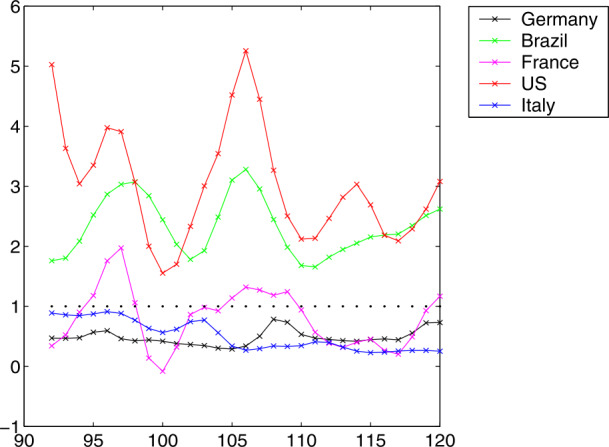
Estimates of $R_{0}$ via the time series $r_{n}$ up to day 120

Figure 5 shows $R_{0}\frac{S_{\mathit{SIR}}}{N}$ estimates via $r_{n}$ for the last four weeks before day 120, i.e. March 21st. Except for the United States and Brazil, all countries were more or less successful in pressing $r_{n}$ below one. In all cases, $S_{\mathit{SIR}}/N$ is too close to one to have any influence. The variation in $r_{n}$ is not due to the decrease in $S_{\mathit{SIR}}/N$, but should rather be attributed to political action. As mentioned above, the estimates for $R_{0}$ by $r_{n}$ are always optimistic.

For the figure, the raw Johns Hopkins data were smoothed by a double action of a $1/4$, $1/2$, $1/4$ filter on the logarithms of the data. This smoother keeps constants and linear sections of the logarithm invariant, i.e. it does not change local exponential behaviour. This smoothing was not applied to Fig. 4. It was by far not strong enough to eliminate the apparent 7-day oscillations that are frequent in the Johns Hopkins data, see Fig. 5, Data from the Robert Koch Institute in Germany have even stronger 7-day variations.

#### Properties of the Model

As long as $r_{n}$ is roughly constant, the above approach will always model an exponential outbreak or decay, but never a peak, because the difference equations are linear. It can only help the user to tell if there is a peak ahead or behind, depending on $r_{n}\approx R_{0}$ being larger or smaller than 1. If $r_{n}$ is kept below one, the Confirmed Infectious will not increase, causing no new threats to the health system. Then the $S/N$ factor will not decrease substantially, and a full SIR model is not necessary.

As long as countries keep $r_{n}$ clearly below one, e.g. below 1/2, this would mean that $R_{0}\approx r_{n}\frac{N}{S_{\mathit{SIR}}}$ stays below one if $S_{\mathit{SIR}}\geq N/2$, i.e. as long as the majority of the population has not been in contact with the SARS-CoV-2 virus. This is good news. But observing a small $r_{n}$ can conceal a situation with a large $R_{0}$ if $S_{\mathit{SIR}}/N$ is small. This is one reason why countries need to get a grip on the Susceptibles nationwide.

So far, the above argument cannot replace a SIR model. It only interprets the available data. However, monitoring the Johns Hopkins data in the above way will be very useful when it comes to evaluate the effectively of certain measures taken by politicians. It will be highly interesting to see how the data of Fig. 5 continue, in particular when countries relax their contact restrictions.

### Extension Towards a SIR Model

For cases where one still has to expect $R_{0}>1$, e.g. US and Brazil on day 120 (see Fig. 5), the challenge remains to predict a possible peak. Using the estimates from the previous section is impossible, because they concern the sub-population of Confirmed and are systematically underestimating $R_{0}$. The “real” SIR model will have different parameters, a possibly large amount of undetected Infectious, and it needs the Susceptibles to model a peak and to make the $r_{n}$ estimates realistic.

For an unrealistic scenario, consider *Total Registration*, i.e. all Infected are automatically confirmed. Then the Susceptibles in the Johns Hopkins model would be $S_{n}=N-C_{n}=N-I_{n}-R_{n}-D_{n}$. Now the estimate for $R_{0}$ must be corrected to

$$ r_{n}\displaystyle {\frac{N}{S_{n}} }=r_{n}\displaystyle { \frac{N}{N-C_{n}} }=r_{n}\left (1+\displaystyle { \frac{C_{n}}{N-C_{n}} }\right ) $$

but this change will not be serious during an early outbreak.

If the time series $\beta _{n}=b_{n}\frac{N}{S_{n}}=b_{n}\frac{N}{N-C_{n}}$ for $\beta $ and $\gamma _{n}$ for $\gamma $ are boldly used as predictors for $\beta $ and $\gamma $ in a SIR model, and if the model is started using $S_{n}=N-C_{n}=N-I_{n}-D_{n}-R_{n}$ in the discretised form

$$ \textstyle\begin{array}{rcl} S_{n+1}-S_{n}&=& -\beta \displaystyle {\frac{S_{n}}{N} }I_{n}, \\ I_{n+1}-I_{n}&=& +\beta \displaystyle {\frac{S_{n}}{N} }I_{n}- \gamma I_{n}, \\ (R+D)_{n+1}-(R+D)_{n}&=&-\gamma I_{n}, \end{array} $$

one gets a crude prediction of the peak in case $R_{0}=\beta /\gamma >1$.

**Fig. 6:**
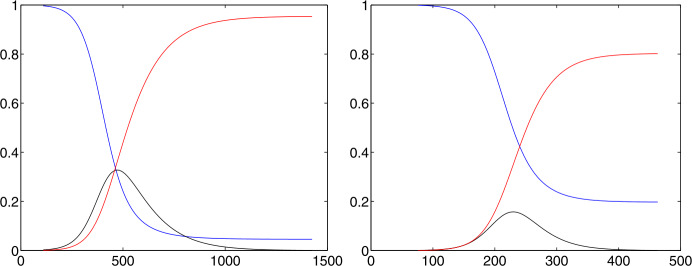
Brute force SIR modelling for US and Germany using last week’s data, at days 109 and 75, with $R_{0}=3.22$ and $R_{0}=2.01$, respectively

Fig. 6 shows results for two cases. The top shows the United States, using data from day 109 (May 10th) and estimating $\beta $ and $\gamma $ from the data one week before. The peak is predicted at day 473 (May 9th, 2021) with a total rate of 33% Infectious, i.e. about 124 million people. With an infection fatality rate of 0.5%, this means about 600,000 casualties in the two weeks around the peak. To see how crude the technique is, the second plot shows Germany using data up to day 75 (April 6th, 2020), i.e. before the peak, and the peak is predicted at day 230 (Sept. 8th, 2020) with about 16% Infected. This would imply about 65,000 casualties around the peak. At day 75, $R_{0}$ was estimated at 2.01, but a few days later the estimate went below 1 (Fig. 5) by political intervention changing $b_{n}$ considerably. See Fig. 10 for a much better prediction using data only up to day 67.

## Extended SIR Model

To get closer to reality, one should combine the data-oriented Johns Hopkins Data Model with a SIR model that accounts for what happens outside of the Confirmed. We introduce the time series

$S$
for the Susceptibles like in the SIR model,
$M$
for the Infectious, not yet confirmed, ($M$ standing for *mysterious*),
$H$
for the unconfirmed Recovered ($H$ standing for *healed*).

This implies that all deaths occur within the Confirmed, though this is a highly debatable issue. It assumes that persons with serious symptoms get confirmed, and nobody dies of COVID-19 without prior confirmation.

### The Hidden Model

The Removed from the viewpoint of a global SIR model including $H$ and $M$ are $H+C$, and thus the SIR model is

20 $$ \textstyle\begin{array}{rcl} S_{n+1}-S_{n}&=& -\beta \displaystyle {\frac{S_{n}}{N} }M_{n}, \\ M_{n+1}-M_{n}&=& \beta \displaystyle {\frac{S_{n}}{N} }M_{n} - \gamma M_{n}, \\ (H+C)_{n+1}-(H+C)_{n} &=& \gamma M_{n}. \end{array} $$

To run this *hidden* model with constant $N=S+M+H+C$, one needs initial values and good estimates for $\beta $ and $\gamma $, which are not the ones of the Johns Hopkins Data Model of Sect. 3.3. We need other ways to get them.

### The Observable Model

The Johns Hopkins variables $D$ and $R$ are linked to the hidden model via $C=I-R-D$. They follow an *observable* model

21 $$ \textstyle\begin{array}{rcl} I_{n}&=&C_{n}-R_{n}-D_{n}, \\ D_{n+1}-D_{n}&=& \gamma _{\mathit{iCD}} I_{n}, \\ R_{n+1}-R_{n}&=& \gamma _{\mathit{iCR}} I_{n} \end{array} $$

with *instantaneous case death and recovery rates* $\gamma _{\mathit{iCD}}$ and $\gamma _{\mathit{iCR}}$ for the Confirmed Infectious. These rates can be estimated separately from the available Johns Hopkins data, and we shall do this below. We call these rates *instantaneous*, because they artificially attribute the new deaths or recoveries at day $n+1$ to the Infectious of the previous day, not of earlier days. They are *case* rates, because they concern the Confirmed. The difference between standard and instantaneous case rates will be treated in Sects. 4.3.1 and 4.3.2.

The observable model is coupled to the hidden model only by $C_{n}$. Any data-driven $C_{n}$ from the observable model can be used to enter the $H+C$ variable of the hidden model, but in an unknown ratio. Conversely, any version of the hidden model produces $H+C$ values that do not determine the $C$ part. Summarising, there is no way to fit the hidden model to the data without additional assumptions.

Various possibilities were tried to connect the Hidden to the Observable. Two will be presented now.

### Fatality Rates

#### Infection Fatality Rate

Recall that the parameter $\gamma _{\mathit{iCD}}$ in the observable model (21) relates case fatalities to the confirmed Infectious of the previous day. In contrast to this, the *infection fatality rate* in the standard literature, denoted by $\gamma _{\mathit{IF}}$ here, is relating to the infection directly, independent of the confirmation, and gives the probability to die of COVID-19 after infection with the SARS-CoV-2 virus, whatever the delay between infection and death is. It was estimated as $\gamma _{\mathit{IF}}=0.56$% by an der Heiden/Buchholz [9] and 0.66% by Verity et al. [21], but specialised for China. Recent data of Streeck et al. [20] gives a value of 0.36% for the Heinsberg population in Germany. For the UK, Ferguson et al. [5] arrive at 0.9%. We shall later use 0.5% for our predictions. But it is very desirable to get more information on infection fatality rates, in particular for different countries. So far, we use a single value globally.

The idea to use the infection fatality rate for information about the hidden system comes from Bommer/Vollmer [1]. The infection fatality rate will be used below in (25) and (27) together with case fatality rates that we consider next.

#### Estimation of Case Fatality Rates

We now focus on probabilities to die either after an infection or after confirmation of an infection. The first is the infection fatality rate given in the literature, but what is latter, the *case fatality rate* $\gamma _{\mathit{CF}}$ when using the Johns Hopkins data? It is clearly not the $\gamma _{\mathit{iCD}}$ in (21), giving the ratio of new deaths at day $n+1$ as a fraction of the confirmed Infectious at day $n$. The deaths at day $n+1$ must be assigned to various earlier days instead.

Case fatality rates in the literature vary strongly, and they are country-dependent. Countries have different ways to detect cases, and because the mortality is age-dependent, different age structures will have a serious influence. The Robert-Koch-Institute [17] mentions 10.5% for Europe and 4.6% for Germany, while De Brouwer et al. [3] has 10.0% for Italy, 4.0% for China, 6.0% for Spain, and 4.3% worldwide. According to Streeck et al. [20], the current estimate of the case fatality rate in Germany by the World Health Organization (WHO) is between 2.2% and 3.4%.

We cannot clean up these inconsistencies. Instead, we now describe a way to estimate case fatality rates per country from the Johns Hopkins data. The basic idealistic assumption is that COVID-19 diseases end after $k$ days from confirmation with either death or recovery. Let us call this the $k$*-day rule*. Suggested values for $k$ start from 14 days for mild cases (an der Heiden/Buchholz [9] WHO [22]) and go up to 30 days, composed of an incubation time of about 5 days and various values between 11 and 25 days for hospitalisation, depending on the amount of intensive care (an der Heiden/Buchholz [9], Robert Koch-Institut [17], Verity et al. [21], Mohring et al. [16]).

Following Schaback [18], one can estimate the probability to survive on day $k+1$ after confirmation, and this works in a stable way per country, based only on $C$ and $D$, not on the unstable $R$ data. In [18] this approach was used to produce $R$ values that comply with the $k$-day rule, but here we use it for estimating the case fatality.

The basic argument lets the new Confirmed $C_{n}-C_{n-1}$ at day $n$ enter into the new deaths $D_{n+1}-D_{n}$ at day $n+1$ with probability $p_{1}=:q_{1}$, into $D_{n+2}-D_{n+1}$ with probability $p_{2}(1-p_{1})=:q_{2}$ and so on. The rest enters into the new Recovered at day $n+k$ with probability $q_{k+1}$ if we set $p_{k+1}=1$ and define

22 $$ q_{i}=p_{i} \displaystyle {\prod _{j=1}^{i-1}(1-p_{j}),\;1\leq i\leq k+1}. $$

Then the estimated case fatality rate is $1-q_{k+1}$, while the case recovery rate is $q_{k+1}$. Therefore the technique of [18] performs a fit

23 $$ \textstyle\begin{array}{rcl} D_{n}-D_{n-1}&\approx & \displaystyle {\sum _{i=1}^{k}q_{i}(C_{n-i}-C_{n-i-1}) }, \\ \end{array} $$

over all possible probabilities $p_{i}$ with sum 1 connected to the $q_{i}$ by (22). It assigns all new deaths at day $n$ to previous new infections on previous days in a hopefully consistent way, minimising the error in the above formula under variation of the probabilities $p_{i}$ to die on day $i$ after confirmation, and it delivers case fatality and case recovery rates per country. It formally assigns all recoveries to day $k+1$ after confirmation. Before that day, a living Confirmed cannot be declared to be recovered.

At this point, there is a hidden assumption. The change $C_{n+1}-C_{n}$ to the Confirmed is understood as the number of new registered infections, i.e. it is treated like $I_{n+1}-I_{n}$, disregarding short-time death or recovery. But replacing $C_{n-i}-C_{n-i-1}$ by $I_{n-i}-I_{n-i-1}$ in (23) would connect a cumulative function to a non-cumulative function. Furthermore, this requires the unsafe data of the Recovered.

**Fig. 7:**
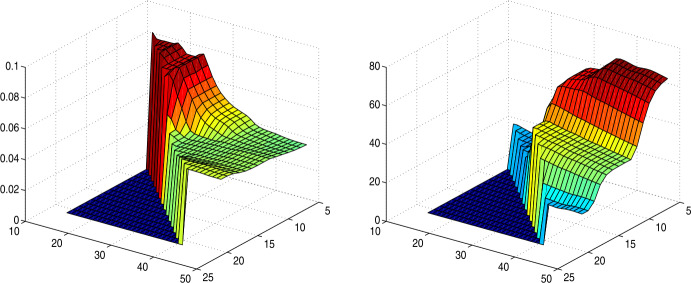
Left: case fatality rate for Germany based on data at day 109, as functions of $k$ (right axis) and the data backlog $B\geq 2k$ (left axis). Right: Root mean-square error for (23)

In fact, the estimation via the fit (23) is unexpectedly reliable, provided one looks at $1-q_{k+1}$ or $q_{k+1}$, not at single probabilities $p_{j}$, and if sufficiently many $n$ are used. This follows from a series of experiments that we do not document fully here, except for Fig. 7. In [18], data for $2k$ days backwards were used for the estimation, and results did not change much when more or less data were used or when $k$ was modified. Here, the range $7\leq k\leq 21$ was tested, and backlogs of up to 50 days from day 109. See Fig. 7 below for an example. It is typical here and for many other cases that a value of $k=14$ performs well, with a backlog of $2k=28$ days for the fit in (23). Using larger $k$ needs a larger backlog, but then the estimation is not time-local enough to produce up-to-date estimates, because outdated values are used. Figure 7 shows the variation of the case fatality rate estimation when $k$ and the backlog are varied. The rates usually do not vary much and have plateaus for $k\geq 14$, but of course the errors decrease when $k$ is taken larger, because there are more days to assign deaths to.

See the first column of Table 1 for estimates of case fatality rates for different countries, calculated on day 109 (May 10th) for $k=14$ and a backlog of 28 days. They comply with the values from the literature cited above. Their interpretation depends strongly on the strategy for confirmation. In particular, they are high when only serious cases are confirmed, e.g. cases that need hospital care. If many more people are tested, confirmations will contain plenty of much less serious cases, and then the case fatality rates are low.

**Table 1:** Case fatality and detection rates, estimated on day 109 using the 14-day rule and a backlog of 28 days

Country	Death rate	Detection rate
Germany	0.047	0.106
Brazil	0.094	0.053
Italy	0.138	0.036
Spain	0.085	0.059
Sweden	0.157	0.032
Austria	0.052	0.096
France	0.122	0.041
UK	0.145	0.035
US	0.067	0.075

The instantaneous case death rate $\gamma _{\mathit{iCD}}$ of (21) for the Johns Hopkins data comes out around 0.004 for Germany on day 109 by direct inspection of the data via

24 $$ \gamma _{\mathit{iCD}}\approx \displaystyle {\frac{D_{n+1}-D_{n}}{I_{n}} }, $$

while the Case Fatality Rate $\gamma _{\mathit{CF}}$ in Table 1 is about 0.047. The deaths have to be attributed to different days using the $k$-day rule, they cannot easily be assigned to the previous day without making the rate smaller.

#### The Detection Rate

A simple way to understand the quotient $\frac{\gamma _{\mathit{IF}}}{\gamma _{\mathit{CF}}}$ of the infection fatality rate $\gamma _{\mathit{IF}}$ and the case fatality rate $\gamma _{\mathit{CF}}$ as a *detection rate* is to ask for the probability $p(C)$ for Confirmation. If the probability to die after Confirmation is $\gamma _{\mathit{CF}}$, and if there are no deaths outside confirmation, then

$$ p(D)=p(C)\cdot p(D|C), $$

by conditional probabilities, and

$$ p(C)=\frac{p(D)}{p(D|C)} = \frac{\gamma _{\mathit{IF}}}{\gamma _{\mathit{CF}}} . $$

See the second column of Table 1, prepared for $\gamma _{\mathit{IF}}=0.005$. The rate depends on good estimates of the infection fatality rate, and the new value 0.0036 by Streeck et al. [20] will decrease the detection rate for Germany from 10.6% to 7.7% for the Heinsberg subpopulation.

All of this is comparable to the findings of Bommer/Vollmer [1] and uses the basic idea from there, but with a somewhat different technique and different results. There, the values were 7% for March 23rd and 9% for March 30th, while Mohring et al. [16] assume 20% on April 29th.

#### Using Fatality Rates for the Hidden Model

If the case fatality rates $\gamma _{\mathit{CF}}$ of Table 1 are used with a known infection fatality rate $\gamma _{\mathit{IF}}$, one should obtain an estimate of the total Infectious. If the formula (23) is written as

$$ \displaystyle {\sum _{i=1}^{k}q_{i}(C_{n-i}-C_{n-i-1}) }\approx D_{n}-D_{n-1} \approx \displaystyle {\sum _{i=1}^{k}\tilde{q}_{i}(S_{n-i-1}-S_{n-i}) } $$

in terms of the previous new infections $S_{n-i-1}-S_{n-i}$ in terms of Susceptibles with daily infection fatality probabilities $\tilde{q}_{i}$, one should maintain

$$ \gamma _{\mathit{CF}}=\displaystyle {\sum _{i=1}^{k} q_{i}} \text{ and } \displaystyle { \gamma _{\mathit{IF}}=\sum _{i=1}^{k} \tilde{q}_{i} }, $$

and this works by setting

25 $$ C_{n}-C_{n-1}=\displaystyle {\frac{\gamma _{\mathit{IF}}}{\gamma _{\mathit{CF}}} } (S_{n-1}-S_{n}) $$

in general, without using the unstable $p_{i}$. This is the first connection of the Observable to the Hidden, namely $C$ to $S$. Like in the discussion following (23) one can argue to use $M$ instead of $S$ in (25), but this would again connect a cumulative variable to a non-cumulative one.

#### Local Estimation of Fatality Rates

Because politicians change testing strategies and the parameters $\beta $ and $\gamma $, the estimation of the Case Fatality Rate should be made locally, not globally. Using the experience of Schaback [18] and Sect. 4.3.2, we shall use a fixed $k=14$ for the $k$-day rule and data for a fixed backlog of $2k$ days. Then the formula (25) has $\gamma _{\mathit{CF}}$ varying with $n$ as far as Johns Hopkins data are available.

### Recovery Rates

We need another parameter to connect the hidden to the observable model. There are many choices, and after some failures we selected the constant $\gamma _{\mathit{iIR}}$ in a model equation

$$ H_{n+1}-H_{n}=\gamma _{\mathit{iIR}}M_{n}. $$

Following what was mentioned about *instantaneous* rates in Sect. 4.2, $\gamma _{\mathit{iIR}}$ is an *instantaneous Infection Recovery Rate*, relating the new unregistered Recovered to the unregistered Infections the day before.

#### Estimation of Recovery Rates

A good value of $\gamma _{\mathit{iIR}}$ can come out of a field experiment that produces time series for $M$ and $H$, i.e. for unregistered Infectious and unregistered Recovered. Then the instantaneous Infection Recovery rate $\gamma _{\mathit{iIR}}$ can be obtained directly by

$$ \displaystyle {\frac{H_{n+1}-H_{n}}{M_{n}} }\approx \gamma _{\mathit{iIR}}. $$

The Infection Recovery rate $\gamma _{\mathit{IR}}=1-\gamma _{\mathit{IF}}$ does not help, because we need an instantaneous rate that has no interpretation as a probability.

With the risk of using unstable data of the Recovered, we can look at the instantaneous Case Recovery rate

26 $$ \displaystyle {\frac{R_{n+1}-R_{n}}{I_{n}} }\approx \gamma _{\mathit{iCR}} $$

that is available from the Johns Hopkins data, and comes out experimentally to be rather stable, provided that countries have useful data for the Recovered. Otherwise, we have to use the technique of Schaback [18] for estimating them. The rate $\gamma _{\mathit{iIR}}$ must be larger than $\gamma _{\mathit{iCR}}$ because we now are not in the subpopulation of the Confirmed, and nobody can die without going first into the population of the Confirmed. As long as no better data are available, we shall use the formula

27 $$ \gamma _{\mathit{iIR}}=\displaystyle {\frac{1-\gamma _{\mathit{IF}}}{1-\gamma _{\mathit{CF}}} } \gamma _{\mathit{iCR}}= \displaystyle {\frac{\gamma _{\mathit{IR}}}{\gamma _{\mathit{CR}}} } \gamma _{\mathit{iCR}}=\displaystyle {\frac{\gamma _{\mathit{iCR}}}{\gamma _{\mathit{CR}}} } \gamma _{\mathit{IR}} $$

that implements two meaningful arguments:

1. the value $\gamma _{\mathit{iCR}}$ is increased by the ratio $\frac{\gamma _{\mathit{IR}}}{\gamma _{\mathit{CR}}}$ of Recovered probabilities for the Infected and the Confirmed,
2. the value $\gamma _{\mathit{IR}}$ is multiplied by a factor $\frac{\gamma _{\mathit{iCR}}}{\gamma _{\mathit{CR}}}$ for transition to immediate rates, and this factor is the transition factor for the Confirmed Recovered.

The above strategy is debatable and may be the weakest point of this approach. However, others turned out to be worse, mainly due to instability of results. On the positive side, the final prediction will not need it, see (32) below. It enters only the intermediate step when $S$, $M$, and $H$ are calculated in the time range of the available Johns Hopkins data, see (28) in Sect. 4.5. And, finally, there is hope that there will be field experiments that yield reliable values directly.

#### Practical Approximation of Recovery Rates

In (27) the rate $\gamma _{\mathit{IR}}$ is fixed, and the rate $\gamma _{\mathit{CR}}$ is determined locally via Sect. 4.3.5. The rate $\gamma _{\mathit{iCR}}$ follows from the time series

$$ \displaystyle {\frac{R_{n+1}-R_{n}}{I_{n}} }\approx \gamma _{\mathit{iCR}} $$

as in (21). This works for countries that provide useful data for the Recovered. In that case, and in others to follow below, we can take the time series itself as long as we have data. For prediction, we estimate the constant from the time series using a fixed backlog of $m$ days from the current day, i.e. we take the mean of the last $m+1$ values. Since many data have a weekly oscillation, due to data being not properly delivered during weekends, the backlog should not be less than 7.

But for certain countries, like the United Kingdom, the data for the Recovered are useless. In such cases, we employ the technique of Schaback [18] to estimate the Recovered using the $k$-day rule and a backlog of $2k$ days, like in Sect. 4.3.5 for the case fatality rates.

### Model Calibration

We now have everything to run the hidden model, but we do it first for days with Johns Hopkins data, delaying predictions to Sect. 5. This is a *calibration step* that leads to estimations of $S$, $M$, and $H$ from the observed data of the Johns Hopkins source, without any need for sophisticated fitting algorithms. With the parameters from above, we use the new relations

28 $$ \textstyle\begin{array}{rcl} C_{n+1}-C_{n}&=&\displaystyle {\frac{\gamma _{\mathit{IF}}}{\gamma _{\mathit{CF}}} } (S_{n}-S_{n+1}), \\ H_{n+1}-H_{n}&=&\gamma _{\mathit{iIR}}M_{n} \end{array} $$

in a specific way. We set up the second model equation in (20) for $M$ as

29 $$ \textstyle\begin{array}{rcl} M_{n+1}-M_{n} &=& S_{n}-S_{n+1}-\gamma _{n} M_{n} \\ &=& \displaystyle {\frac{\gamma _{\mathit{CF}}}{\gamma _{\mathit{IF}}} }( C_{n+1}-C_{n})- \gamma _{n} M_{n} \\ &=& \displaystyle {\frac{\gamma _{\mathit{CF}}}{\gamma _{\mathit{IF}}} }( C_{n+1}-C_{n})-(C_{n+1}-C_{n}+H_{n+1}-H_{n}) \\ &=& \left (\displaystyle {\frac{\gamma _{\mathit{CF}}}{\gamma _{\mathit{IF}}} }-1 \right )( C_{n+1}-C_{n})-\gamma _{\mathit{iIR}}M_{n} \end{array} $$

that can be solved if an initial value $\tilde{M}_{0}$ is prescribed. Then (28) is run to produce the $S_{n}$ and $H_{n}$, with starting values that we describe in Sect. 4.5.1. If $\beta _{n}$ and $\gamma _{n}$ are calculated by

30 $$ \textstyle\begin{array}{rcl} \beta _{n}\displaystyle {\frac{S_{n}}{N} }M_{n}&=&S_{n}-S_{n+1}, \\ \gamma _{n} M_{n} &=& C_{n+1}-C_{n}+H_{n+1}-H_{n}, \end{array} $$

respectively, the balance equation $N=S+M+H+C$ follows from (29) and (30).

#### Starting Values

Since the populations are large, the starting values for $S$ are not important. Beginning at the full population $N$ from a very early day, the $S$ values are calculated from (28) first, just to get values $S_{j}$ for actually starting at later days.

Then the first day $j$ is taken where $C_{j}$ is at least 10, and $k$ days later the start value for $H$ is set as

31 $$ H_{j+k}=C_{j}\displaystyle {\frac{\gamma _{\mathit{CF}}}{\gamma _{\mathit{IF}}} } $$

using the $k$-day rule with $k=14$. This divides the $C_{j}>I_{j}$ value by the detection rate, i.e. roughly all estimated undetected Infectious at time $j$ are assumed to be healed $k$ days later, i.e. at day $j+k$. Then the starting value for $M_{j+k}$ is calculated via the balance equation $N=S+M+H+C$ from the $S_{j+k}$ value calculated by the previous paragraph. Finally, the calibration starts at day $j+k$ by the above formulae. Unfortunately, this is a serious limit preventing application to very short time series.

The starting value for $H$ is irrelevant for $H$ itself, because only differences enter into the model, but it determines the starting value for $M$ due to the balance equation. Anyway, it turns out experimentally that the starting values do not matter much, if the model is started early. The hidden model (20) depends much more strongly on $C$ than on the starting values.

Fig. 10 contains a wide variation of the starting value (31) for $H$ at the starting point, by multipliers between 1/32 and 32. This has hardly any effect on the results, the lines getting somewhat thicker. The variation in starting values get more visible in other cases, see the right-hand plot in Fig. 10 for the United States. But the influence on predictions is negligible.

#### Examples

The figures to follow in Sect. 5.2 show the original Johns Hopkins data together with the hidden variables $S$, $M$, and $H$ that are calculated by the above technique. The calibration runs up to the vertical line where predictions start. Note that the only ingredients beside the Johns Hopkins data are the number $k=14$ for the $k$-day rule, the Infection Fatality Rate $\gamma _{\mathit{IF}}$ from the literature, equations (28), and the backlog of $m=7$ days for estimation of constants from time series.

## Predictions using the Full Model

To let the combined model predict the future, or to check what it would have predicted if used at an earlier day, we take the calibrated model of the previous sections up to a day $n$ and use the values $S_{n}$, $M_{n}$, $H_{n}$, $C_{n}$, $I_{n}$, $R_{n}$ and $D_{n}$ for starting the prediction. With the variable $\mathit{HC}:=H+C$, we use the recursion

32 $$\begin{array}{rlr} S_{i+1} & = & S_{i}-\beta \frac{S_{i}}{N}M_{i},\\ M_{i+1} & = & M_{i}+\beta \frac{S_{i}}{N}M_{i}-\gamma M_{i},\\ {\mathrm{HC}}_{i+1} & = & {\mathrm{HC}}_{i}+\gamma M_{i},\\ C_{i+1} & = & C_{i}+\gamma_{\mathrm{IF}}(S_{i}-S_{i+1})/\gamma_{\mathrm{CF}},\\ R_{i+1} & = & R_{i}+\gamma_{\mathrm{iCR}}I_{i},\\ D_{i+1} & = & D_{i}+\gamma_{\mathrm{iCD}}I_{i},\\ I_{i+1} & = & C_{i+1}-R_{i+1}-D_{i+1},\\ H_{i+1} & = & {\mathrm{HC}}_{i+1}-C_{i+1}. \end{array}$$

This needs fixed values of $\beta $ and $\gamma $ that we estimate from the time series for $\beta _{n}$ and $\gamma _{n}$ by using a backlog of 7 days, following Sect. 4.5. The instantaneous rates $\gamma _{\mathit{iCR}}$ and $\gamma _{\mathit{iCD}}$ can be calculated via their time series, as in (26) and (24), using the same backlog. We do this at the starting point of the prediction, and then the model runs in a *no political change* mode. Examples will follow in Sect. 5.2.

### Properties of the Full Model

The first part of the full model (32) is a standard SIR model for the variables $S$, $M$ and $H+C$, and inherits the properties of these as described in Sect. 2. It does not use the $\gamma _{\mathit{iIR}}$ parameter of the second equation in (28), and it uses the first the other way round, now determining $C$ from $S$, not $S$ from $C$.

The balances $N=S+M+H+C$ and $C=I+D+R$ are maintained automatically, and the time series for $S$, $C$, $R$, $H+C$, and $D$ stay monotonic as long as $M$ and $I$ are non-negative. To check the monotonicity of $H$, consider

$$ \textstyle\begin{array}{rcl} H_{i+1}-H_{i} &=& \mathit{HC}_{i+1}-\mathit{HC}_{i}-C_{i+1}+C_{i} \\ &=& \gamma M_{i}-\displaystyle {\frac{\gamma _{\mathit{IF}}}{\gamma _{\mathit{CF}}} }(S_{i}-S_{i+1}) \\ &=& \left (\gamma -\beta \displaystyle { \frac{\gamma _{\mathit{IF}}}{\gamma _{\mathit{CF}}} }\displaystyle {\frac{S_{i}}{N} } \right )M_{i}. \end{array} $$

The bracket is positive if

$$ R_{0}=\displaystyle {\frac{\beta }{\gamma } }< \displaystyle { \frac{\gamma _{\mathit{CF}}}{\gamma _{\mathit{IF}}} }\displaystyle {\frac{N}{S_{i}} } \geq \displaystyle {\frac{\gamma _{\mathit{CF}}}{\gamma _{\mathit{IF}}} }, $$

which is enough for practical purposes as long as detection rates $\frac{\gamma _{\mathit{IF}}}{\gamma _{\mathit{CF}}}$ are low and $R_{0}$ is not excessively large. Anyway, $H$ should be monitored.

The slopes of $S$ and $C$ are always connected by (25), and those of $R$ and $D$ are connected by

33 $$ R_{i+1}-R_{i}=\displaystyle {\frac{\gamma _{\mathit{iCR}}}{\gamma _{\mathit{iCD}}} }(D_{i+1}-D_{i}) $$

in the prediction part. But the figures below will show logarithms, and therefore the slope parallelism will not be visible.

By Sect. 2.11, the hidden Infectious $M$ will always go to zero, and the variables $S$ and $H+C$ will level out in the long run. Since $C$ is increasing, it must level out as well, and $I$ must level out because $R$ and $D$ do. But due to the equations for $R$ and $D$, the final level of $I$ must be zero.

The asymptotic levels of $S$ and $H+C$ follow from 2.11, but not the interesting level of $D$, the total death toll. If the prediction is started at day $n$, then

$$ R_{\infty }-R_{n}=\displaystyle {\frac{\gamma _{\mathit{iCR}}}{\gamma _{\mathit{iCD}}} }(D_{ \infty }-D_{n}), $$

obtained by summation of (33), connects the asymptotic deaths and confirmed recoveries. From the connection of $S$ and $C$ we likewise get

$$ C_{\infty }-C_{n}=\displaystyle {\frac{\gamma _{\mathit{iIF}}}{\gamma _{\mathit{CF}}} }(S_{n}-S_{ \infty }). $$

With $C_{\infty }=R_{\infty }+D_{\infty }$ we now have three independent equations for the unknowns $C_{\infty }$, $D_{\infty }$, $R_{\infty }$. Because the theory of Sect. 2.11 yields $S_{\infty }$ and $H_{\infty }+C_{\infty }$ in terms of $\beta $ and $\gamma $, we know $S_{\infty }$ and can get $H_{\infty }$ from $C_{\infty }$. But if the simulation is run long enough, one can easily read the asymptotic values off the plots.

### Examples of Predictions

**Fig. 8:**
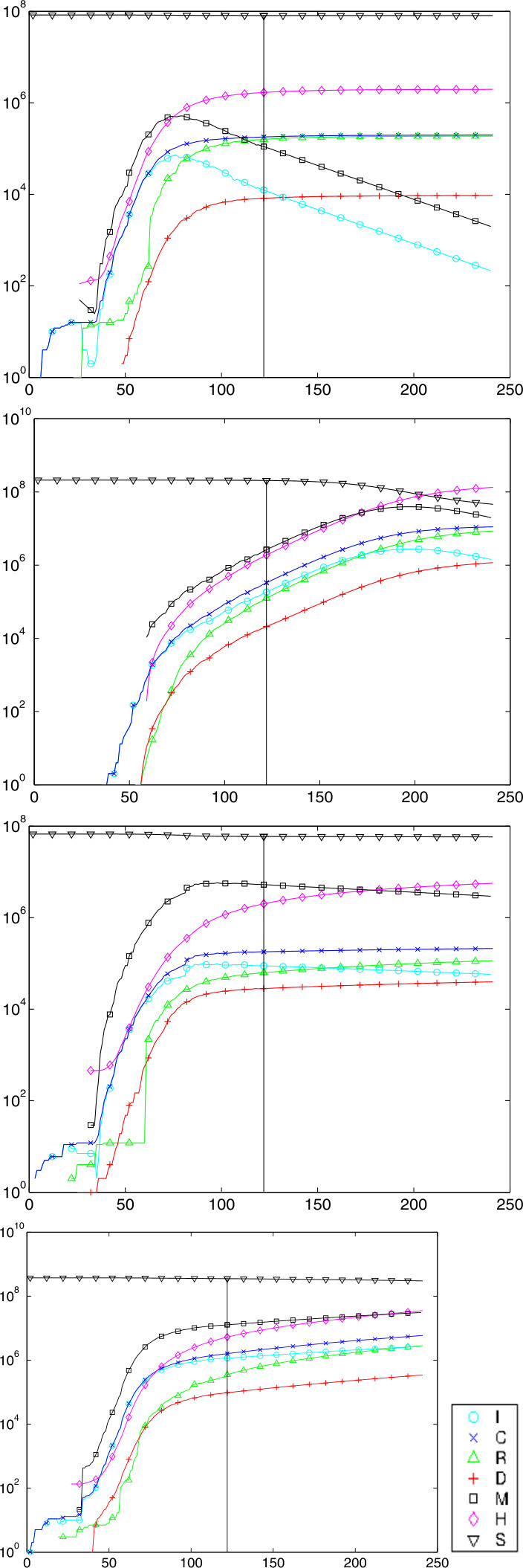
Predictions for countries Germany, Brazil, France, and US on day 122 marked by the vertical line. The $S$, $M$, $H$ values to the left are obtained by calibration, the $C$, $R$, $D$, $I$ values there are the original Johns Hopkins data. Not all data points have marks

Figure 8 shows predictions on day 122, May 23rd, for Germany, Brazil, France, and USA, from the top. The plots for countries behind their peak are rather similar to those for Germany and France. The other two countries are selected because they still have to face their peak, if no action is taken to change the parameters.

The plots show that Germany can expect to get away with no more than 10000 casualties in the long run, while Brazil goes for a peak of about 20 million hidden Infectious in fall 2020 ($M$, symbol $\Box $) and a final death toll of about 1 million ($D$, symbol +). The United States would have to face a peak of hidden Infectious of about 25 million in mid-January 2021, and more than 1 million COVID-19 deaths in October 2021, and still rising. But of course, these predictions assume that reality follows the model and that there are no parameter changes by political action.

The estimated $R_{0}$ values are 0.65, 2.19, 0.42, and 1.75, respectively. Note that these are not directly comparable to Fig. 5, because they are the fitted constants to the backlog of a week, and using (30) instead of (19), avoiding the systematic underestimation of the latter. The hidden $M$ and $H$ (symbols $\Box $ and ⋄) follow roughly the observable $I$ and $C$ (symbols $O$ and $x$), but with a factor due to the detection rate that is different between countries, see Table 1. To enhance visibility, not all data points in the plots are marked with symbols. The $C$, $R$, $I$ and $D$ data left of the vertical line are the original Johns Hopkins data. The $S$, $M$, $H$ data there are calculated by Sect. 4, while to the right the data are predictions for all variables by the full model (32).

All test runs were made for the infection fatality rate $\gamma _{\mathit{IF}}=0.005$, the delay $k=14$ for estimating case fatalities, and a backlog of 7 days when estimating constants out of recent values of time series. The choice $\gamma _{\mathit{IF}}=0.005$ is somewhat between 0.56% from an der Heiden/Buchholz [9], 0.66% from Verity et al. [21], and 0.36% from Streeck et al. [20]. New information on infection fatality rates should be included as soon as they are available, and if possible per country, not global.

When used within estimation routines, the Johns Hopkins data were smoothed by a double application of the 1/4, 1/2, 1/4 filter on the logarithms, like for Fig. 5. But the plots show the original Johns Hopkins and prediction data.

### Evaluation of Predictions

**Fig. 9:**
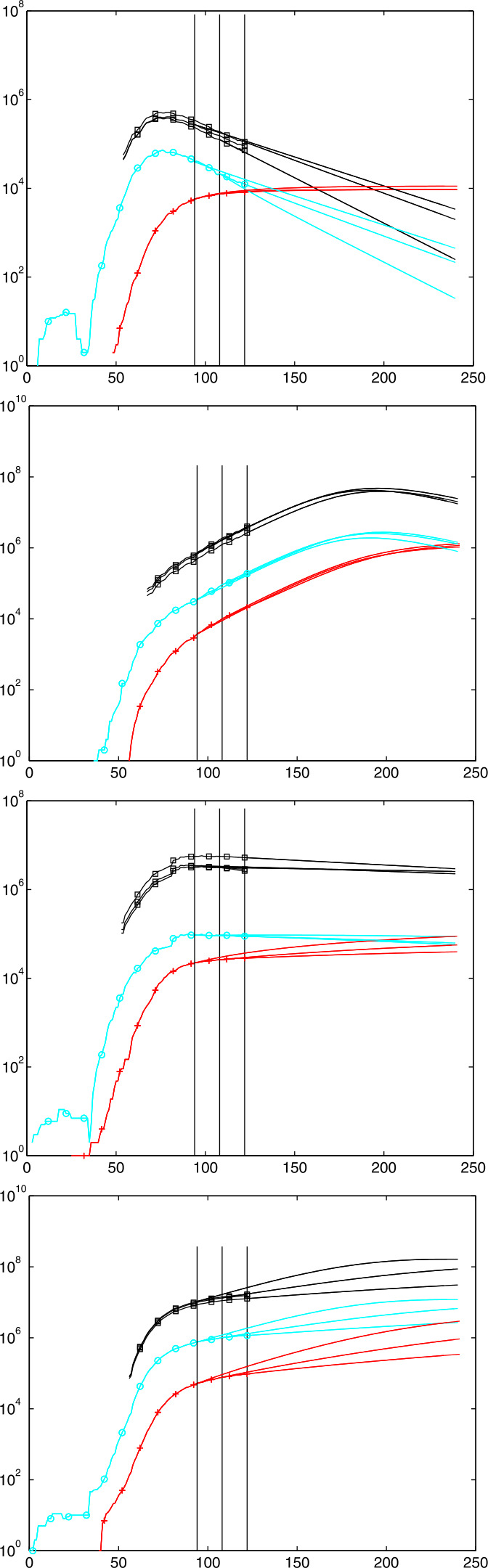
Predictions for countries Germany, Brazil, France, and USA on days 122, 108, and 94, marked by vertical lines. Legend as in Fig. 8, but only $M$, $I$, and $D$ shown ($M=\Box $, $I = \text{O}$, $D=+$)

To evaluate the prediction quality, one should go back and start the predictions on earlier days, to compare with what happened later. Figure 9 shows over-plots of predictions for days 94, 108, and 122, each a fortnight apart, though there may be parameter changes in the meantime. The starting points of the predictions are marked by vertical lines again. For better visibility, only the death count $D$ (symbol +) and the two non-cumulative variables $M$ and $I$ for the hidden and confirmed Infectious (symbols $\Box $ and $O$) are shown. In particular, the case fatality rates and detection rates of Table 1 change with the starting point of the prediction, and they determine $S$, $M$, and $H$ in the calibration step of Sect. 4.5. This is why the $S$, $M$, and $H$ values differ left of the starting points.

The leftmost prediction on day 94 roughly matches the data available up to day 122 in all cases. It has to be taken into account that errors in such models must proliferate exponentially, and then linearly in logarithmic plots. One can see that the Brazil parameters do not change much, while the three predictions for the United States get better. This might be used to assess effectivity of administrative efforts to handle the pandemics.

**Fig. 10:**
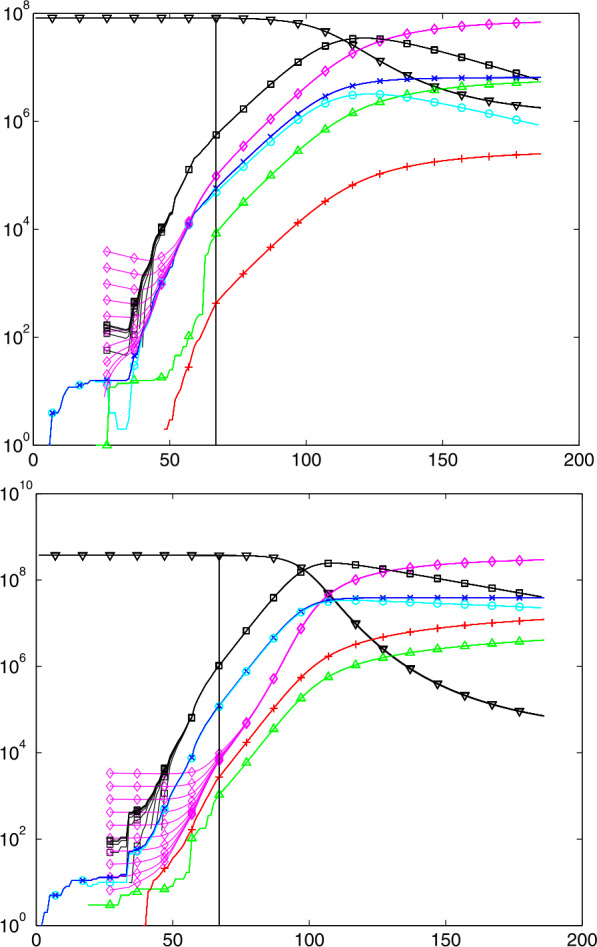
Predictions for Germany and USA on day 67, March 27th, with varying starting values. Legend as in Fig. 8

For an early case in Germany, Fig. 10 shows the prediction based on data of day 67, March 27th. The peak of about 35 million hidden and 3.2 million confirmed Infected is predicted on day 121, May 22nd, with about 82,000 casualties at the peak and about 250,000 finally. A good reason to act politically. Note that the real death count is about 8300 on May 23rd, and the prediction of the day, in Fig. 8, targets a final count of below 10,000.

Quantitative commitments to predictions are rare in the literature, except for rough estimations of dramatic outbreak scenarios. On April 3rd, after the last public restrictions in Germany of March 22nd, 2020, Germany had 1107 deaths and Khailaie et al. [13] predicted “an order of 10,000 deaths” for the next four weeks. This model predicts 15,500 for May 3rd when run on data of April 3rd, while the true deaths were 6812 on May 3rd, after the interventions worked.

On March 16th, day 54, Ferguson et al. [5] predicted deaths in the order of 250,000 in Great Britain, and 1.1 to 1.2 million in the USA “in the most effective mitigation strategy examined”, but not based on the data of the day. In an “unmitigated epidemic” 520,000 deaths in the UK and 2.2 million in the US were predicted, under assumption of $R_{0}=2.4$ and a range of $R_{0}$ tested between 2.2 and 2.6. Unfortunately, the model (32) cannot be safely run on day 54 for these countries because there are not enough reliable backlog data. The model can be run if the amount of data used is cut down by choosing $k=7$ for the $k$-day rule. Then the predictions on day 54 are more than 30 million deaths for the US and 801,000 for the UK, with a data-based estimation of $R_{0}=6.06$ for the USA and 4.55 for the UK. There is no reasonable data-driven estimate for $R_{0}$ that comes close to $R_{0}=2.4$ used by Ferguson et al. [5] for both countries. They had a much more serious outbreak than assumed by Ferguson et al. on March 16th. See Fig. 5 for much later data-based estimates for the US that still are very large.

The use of the Infection Fatality Rate is somewhat different from Streeck et al. [20] and Bommer/Vollmer [1], but results are similar. If the rate 0.0036 of [20] is used in a test run based on data of May 2nd, the estimated number $M_{n}+C_{n}$ of total Infected comes out as 1.7 million, while [20] gets 1.8 million by the formula $D_{n}/0.0036$ for the same day.

The parameter changes by political measures turned out to be rather effective, like in many countries that applied similar strategies. But since parts of the population want to go back to their previous lifestyle, all of this is endangered, and the figures should be monitored carefully.

Of course, all of this makes sense only under the assumption that reality follows the model, in spite of all attempts to design a model that follows reality.

## Conclusion and Open Problems

**Fig. 11:**
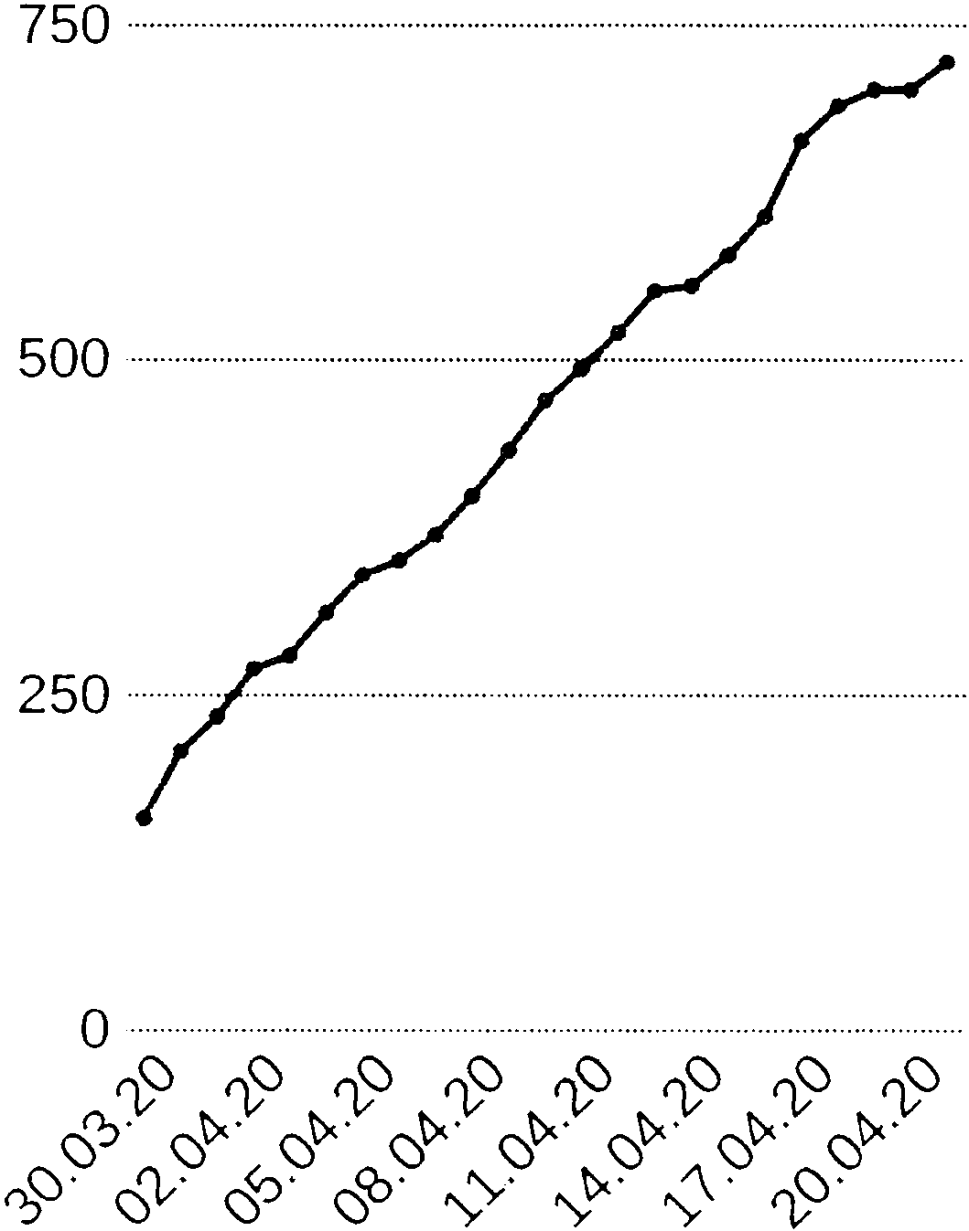
Infectious in Göttingen city and county, as of April 22nd, 2020 in the local newspaper “Göttinger Tageblatt”. No exponential outbreak

So far, the model presented here seems to be useful, combining theory and practically available data. It is data-driven to a very large extent, using only the infection fatality rate from outside for prediction, and the approximation (27) for calibration. On the downside, there is quite a number of shortcomings:

- Like the data themselves, the model needs regular updating. As far as the Johns Hopkins data are concerned, the model updates itself by using the latest data for its internal parameter estimation, but it needs changes as soon as new information on the hidden infections come in.
- There may be better ways of estimating the hidden part of the epidemics. However, it will be easy to adapt the model to other parameter choices. If time series for the unknown variables get available, the model can easily be adapted to being data-driven by the new data.
- The treatment of delays is unsatisfactory. In particular, infected persons get infectious immediately, and the $k$-day rule is not followed at all places in the model. But the rule is violated as well in the data (Schaback [18]).
- There is no stochastics involved, except for simple things like estimating constants by means, or for certain probabilistic arguments on the side, e.g. in Sect. 4.3.2. But it is not at all clear whether there are enough data to do a proper probabilistic analysis.
- As long as there is no probabilistic analysis, there should be more simulations under perturbations of the data and the parameters. A few were included, e.g. for Sect. 4.3.2 and Figs. 7 and 10, but a large number was performed in the background when preparing the paper, making sure that results are stable. However, there are never too many test simulations.
- Totally different models were not considered, e.g. the classical ones with delays (Hoppenstaedt/Waltman [10, 11]), and agent-based approaches (Ferguson et al. [5]) that model infections via contacts and can care for spatial distributions.
- The model needs quite an amount of backward data, making it useless at the very beginning of an outbreak.
- Under certain circumstances, epidemics do not show an exponential outbreak, in particular if they hit only locally and a prepared population. See Fig. 11 for the COVID-19 cases in Göttingen and vicinity.

## Supplementary Information

Below is the link to the electronic supplementary material.

(ZIP 256 kB)

## Footnotes

1 Original text in German, April 16th: *Schnelle Modelle, die dem Abgleich mit der Wirklichkeit standhalten, sind eine wichtige Voraussetzung, das Land politisch durch die Seuche zu steuern.*
