# Correction to: On COVID-19 Modelling

**DOI:** 10.1365/s13291-021-00240-6

**Published:** 2021-09-06

**Authors:** Robert Schaback

**Affiliations:** Institut für Numerische und Angewandte Mathematik, Universität Göttingen, Lotzestraße 16-18, 37083 Göttingen, Germany

**Correction to: Jahresber Dtsch Math-Ver (2020) 122:167–205** 10.1365/s13291-020-00219-9

The article “On COVID-19 Modelling” written by Robert Schaback was originally published online first on 14. April 2020 without “Open Access”. After this publication the author decided to opt for Open Choice and to make the article an Open Access publication. Therefore the copyright of the article has been changed on 7 July 2021 to © The Author(s) 2020 and the article is forthwith distributed under a Creative Commons Attribution.

This article is licensed under a Creative Commons Attribution 4.0 International License, which permits use, sharing, adaptation, distribution and reproduction in any medium or format, as long as you give appropriate credit to the original author(s) and the source, provide a link to the Creative Commons licence, and indicate if changes were made. The images or other third party material in this article are included in the article’s Creative Commons licence, unless indicated otherwise in a credit line to the material. If material is not included in the article’s Creative Commons licence and your intended use is not permitted by statutory regulation or exceeds the permitted use, you will need to obtain permission directly from the copyright holder. To view a copy of this licence, visit http://creativecommons.org/licenses/by/4.0/.

